# A 26-year old young male with severe anemia

**DOI:** 10.1016/j.rmcr.2021.101575

**Published:** 2022-01-05

**Authors:** Muhammad Salick, Raghav Chaudhary, Fabiana Maria Robledo, Praveen B. Datar, Arkar Htoo, Boris Shkolnik, Woon Hean Chong, Amit Chopra, Biplab K. Saha

**Affiliations:** a Department of Pulmonary and Critical Care Medicine, Albany Medical Center, Albany, NY, USA; b Department of Medicine, Ozarks Medical Center, West Plains, MO, USA; c Department of Nephrology, Albany Medical Center, Albany, NY, USA; d Department of Pulmonary and Critical Care Medicine, Ozarks Medical Center, West Plains, Missouri, USA; e Department of Pathology, Albany Medical Center, Albany, NY, USA; f Department of Intensive Care Medicine, Ng Teng Fong General Hospital, National University Health System, Singapore City, Singapore

**Keywords:** Anemia, Choriocarcinoma, Lung mass, Testicular mass

## Abstract

Testicular choriocarcinoma is a subset of Non-Seminomatous Germ Cell Tumors (NSGT) which is considered the rarest and most aggressive testicular cancer. It primarily affects males between the ages of 25–30 years. Unlike other testicular neoplasms that carry a cure rate of 95%, choriocarcinoma has significantly lower rate of cure. Therefore, early detection and prompt treatment is necessary to improve survival. We present an unusual case of Choriocarcinoma presenting as severe anemia along with distant metastases to lung and brain. We also discuss diagnostic approach and treatment challenges in patients with Choriocarcinoma.

## 1 Introduction

Choriocarcinoma is the most aggressive and rarest subtype of non-seminomatous Germ Cell Tumors (NSGCT) comprising of only 1%–3% of all testicular cancers [1]. Commonly choriocarcinoma presents with testicular mass with evidence of distant metastasis to liver, lung, and brain. Severe anemia as an initial presentation has not been described. We report a unique case of choriocarcinoma presenting as severe anemia along with distant metastasis to lung and brain.

## 2 Case report

A 26-year-old male presented to the hospital with progressive dyspnea and fatigue for one week. The patient had no past medical history. He denied any fever, night sweats, chills, weight loss, hematemesis, melena, cough, sputum production, orthopnea or paroxysmal nocturnal dyspnea. He was a nonsmoker and was not taking any medications at home regularly. He also denied any history of recreational drug use. His vital signs were as follows: heart rate, 122 beats/minute, BP 130/66, respiratory rate of 25, oxygen saturation 96% on room air and temperature of 37.2C. Physical examination revealed a tired looking young man in no acute distress. He had severe conjunctival pallor but no evidence of central cyanosis. The cardiopulmonary examination was normal. Abdominal palpation demonstrated slightly enlarged liver.

**Fig. 1:**
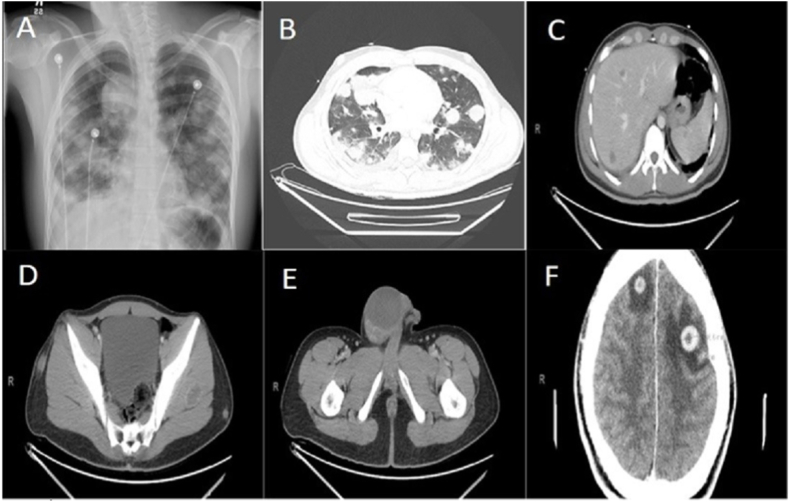
A: Bilateral diffuse rounded opacities with predominant with predominant right supra-hilar mass. B: CT chest with contrast showed bilateral lung nodule and masses with predominant mass in right upper lobe/mediastinum. C: CT abdomen showed hepatic and splenic metastases. D: CT Pelvis showed left gluteal mass, which was biopsied. E: CT pelvis showed homogenous right testicular mass. F: CT head showed bilateral hemorrhagic brain metastases.

Laboratory examination showed white cell count 21,700/μL, Hemoglobin 4.1 mg/dl, hematocrit 19.1% and platelet count 371000/μL. Iron studies revealed total iron 24 UG/DL, Total iron binding capacity 180UG/dL and iron saturation of 13%. Chest radiograph showed diffuse bilateral large pulmonary nodules and right peri-hilar mass (Fig. 1A). Computed tomography (CT) scan of chest, abdomen and pelvis showed bilateral pulmonary, hepatic and splenic nodules along with para-spinal, para-aortic, inguinal lymphadenopathy, right Gluteus Maximus soft tissue mass and homogenous right testicular mass (Fig. 1B–E). CT head with contrast was obtained as a part of metastatic work up that showed 2 bilateral frontal cortex enhancing lesions with internal hemorrhage and surrounding vasogenic edema (Fig. 1F). Tumor markers showed extremely high levels of Beta Human Chorionic Gonadotrophin (β-hcg) 180326 mIU/ml (normal <2.0 mIU/ml) and AFP of 3 ng/ml (normal 0–9.0 ng/ml).

**Fig. 2:**
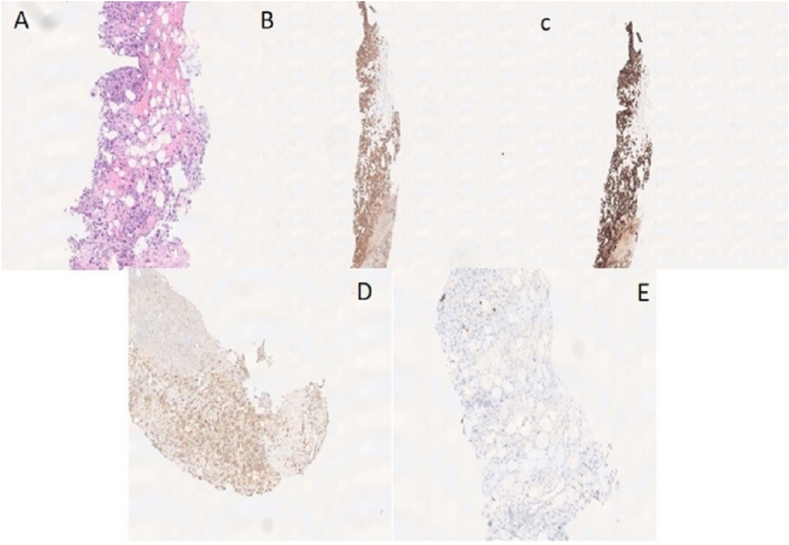
A: H&E core biopsy of the right hip soft tissue mass showed mixture of Cytotrophoblasts and multinucleated Syncytiotrophoblasts in the background of significant hemorrhage and necrosis. B: Strong positive staining for AE1/AE3.C: Strong positive staining for CAM 5.2. D: Rare tumor cells stained with SALL-4. E: Tumor cells stained with C-Kit (CD-117).

The patient was thought to be suffering from metastatic testicular cancer. He underwent CT guided core needle biopsy of the right gluteal soft tissue lesion. Histopathologic analysis showed malignant germ cell tumor, consistent with choriocarcinoma (immunohistochemical stain positive for AE1/AE3, C-Kit, and Cam-5.2, with rare cells stained with SALL-4 and PLAP, and is negative for OCT3-4, AFP, CD30, S-100, and SOX-10) (Fig. 2).

The patient was diagnosed with stage IIIC GCT (choriocarcinoma). He was started on chemotherapy with VIP (Etoposide, Ifosfamide, and Cisplatin). Patient received whole brain radiation with 3000Gy in 10 divided fractions.Treatment course was complicated by development of hemoptysis requiring high flow nasal cannula oxygen support. Also the number and size of CNS metastases increased with internal hemorrhages seen on repeat MRI. Fortunately no complications related to intracranial bleed were encountered. He has been discharged and is currently getting outpatient chemotherapy and remaining radiation cycles. He is alive.

## 3 Discussion

Choriocarcinoma is a rare type of testicular cancer, with peak incidence between ages of 25–30 years [1]. Choriocarcinomas are mostly unilateral and secrete high levels of β-hcg often >10000IU. It spreads rapidly via hematogenous route, therefore widespread metastases are often found at the time of diagnosis. Most common site of metastases is lung, liver, and brain [[2], [3], [4]]. Our case closely resembled the typical clinical presentation. Widespread metastases were found at the time of diagnosis, and his symptoms became noticeable over the period of only 1 week. However, our patient was also unique, as he presented with severe anemia which appeared to be secondary to anemia of chronic disease.

Interestingly, choriocarcinoma metastases have high propensity to bleed and these complications can be rapidly fatal [5,6]. Our patient had hemorrhagic CNS metastasis which increased in size and number during few days of hospitalization. Lung is the most common site of choriocarcinoma metastases and high volume pulmonary metastases can result in life threatening pulmonary hemorrhage, hemothorax and respiratory failure [7]. Our case had significant burden of disease in lungs and during the course of hospitalization developed hemoptysis and high oxygen requirement. Excessive vascular invasion and rapid growth results in tumor outgrowing its blood supply which leads to necrosis and ulceration causing hemorrhagic complications [8]. Respiratory failure in our patient was in part due to Tumor Lysis Syndrome (TLS) as well. Therefore a clinician has to be careful because hemorrhagic complications can happen before and during the chemotherapy due to rapid cell death, and TLS is well-recognized complication in this solid malignancy [9]. Presence of intracerebral metastases form choriocarcinoma is a poor prognostic sign [10].

Choriocarcinomas are biphasic tumors containing both Syncytiotrophoblasts and Cytotrophoblasts. Histopathological diagnoses are based on morphologic evidence of Cytotrophoblast and Syncytiotrophoblast. They usually present with abundant hemorrhage and necrosis and have vascular invasion, ending up with early and disseminated metastasis [11]. However, metastases are unlikely to present with bulky anterior mediastinal mass. Histology as well as immunophenotype in both primary and metastatic disease are similar. Immunohistochemical stains can identify a mixture of cytotrophoblasts, which stain positive with p63 and negative with HPL and intermediate trophoblasts stain negative with p63 and weak positive or negative with HPL weak in the columns of mononucleated cells [3]. Many of these metastatic tumors may have undergone regression in other words “burned-out” [12].

Treatment is usually Bleomycin based except in patients with high burden of pulmonary metastasis to avoid pulmonary hemorrhage and respiratory failure. Beta-HCG is commonly followed for treatment response. Plateau <10mIU/ml indicate a satisfactory response whereas higher plateau usually indicates treatment failure [13,14]. Early treatment is associated with improved outcome. Therefore, in patients with mediastinal mass or testicular mass, and markedly elevated levels of β-hCG (>50,000 MIU/ML), empiric chemotherapy should be administered immediately to improve survival [1]. Prognosis is worse as compared to other testicular cancers, with 5-year survival of pure choriocarcinoma being less than 85% at best [1].

## 4 Conclusion

Distant metastases in young individuals with testicular or mediastinal mass should raise concern for NSGTs. Concomitant findings of hemorrhagic metastases and bHCG >50,000 MIU/ML is highly suggestive of choriocarcinoma. Severe anemia is an unusual and rare presentation of NGTs. Treatment should be instituted as early as possible as a delay in treatment is associated with worse outcomes. Unfortunately, the prognosis of choriocarcinoma is worst among all the testicular cancers.

## Author contribution

All authors were involved in the planning, collection of data, preparation of the initial and final manuscript.

## Declaration of competing interest

The authors have no conflict of interest to disclose.

## References

[1] Reilley M.J., Pagliaro L.C. (2015). Testicular choriocarcinoma: a rare variant that requires a unique treatment approach. Curr. Oncol. Rep..

[2] Chen X., Xu L., Chen X. (2010). Testicular choriocarcinoma metastatic to skin and multiple organs. Two case reports and review of literature. J. Cutan. Pathol..

[3] Alvarado-Cabrero I., Hernández-Toriz N., Paner G.P. (2014). Clinicopathologic analysis of choriocarcinoma as a pure or predominant component of germ cell tumor of the testis. Am. J. Surg. Pathol..

[4] Smith Z.L., Werntz R.P., Eggener S.E. (2018). Testicular cancer: epidemiology, diagnosis, and management. Med. Clin..

[5] Das S., Badhe B., Bibi A. (2015). Sudden death as a complication of choriocarcinoma. J. Forensic Sci..

[6] Syed S., Westwood A.J. (2013). Clinical reasoning: a 25-year-old man with headaches and collapse. Neurology.

[7] Tatokoro M., Kawakami S., Sakura M. (2008). Successful management of life-threatening choriocarcinoma syndrome with rupture of pulmonary metastatic foci causing hemorrhagic shock. Int. J. Urol. Off J. Jpn. Urol. Assoc..

[8] Motzer R.J., Bosl G.J. (1987). Hemorrhage: a complication of metastatic testicular choriocarcinoma. Urology.

[9] Kawai K., Takaoka E.-I., Naoi M. (2006). A case of metastatic testicular cancer complicated by tumour lysis syndrome and choriocarcinoma syndrome. Jpn. J. Clin. Oncol..

[10] Oechsle K., Bokemeyer C. (2011). Treatment of brain metastases from germ cell tumors. Hematol. Oncol. Clin. N. Am..

[11] Al-Obaidy K.I., Idrees M.T. (2021). Testicular tumors: a contemporary update on morphologic, immunohistochemical and molecular features. Adv. Anat. Pathol..

[12] Ulbright T.M. (2005). Germ cell tumors of the gonads: a selective review emphasizing problems in differential diagnosis, newly appreciated, and controversial issues. Mod. Pathol. Off J. U S Can. Acad. Pathol. Inc..

[13] Einhorn L.H., Williams S.D., Chamness A. (2007). High-dose chemotherapy and stem-cell rescue for metastatic germ-cell tumors. N. Engl. J. Med..

[14] Lewin J., Dickinson M., Voskoboynik M. (2014). High-dose chemotherapy with autologous stem cell transplantation in relapsed or refractory germ cell tumours: outcomes and prognostic variables in a case series of 17 patients. Intern. Med. J..

